# Role of inducible nitric oxide (iNOS) and nitrosative stress in regulating sex differences in secondary lymphedema

**DOI:** 10.3389/fphys.2024.1510389

**Published:** 2024-12-03

**Authors:** Adana-Christine Campbell, Kevin G. Kuonqui, Gopika Ashokan, Jonathan Rubin, Jinyeon Shin, Bracha L. Pollack, Arielle Roberts, Ananta Sarker, Hyeung Ju Park, Raghu P. Kataru, Andrea V. Barrio, Babak J. Mehrara

**Affiliations:** Plastic and Reconstructive Surgery, Department of Surgery, Memorial Sloan Kettering Cancer Center, New York, NY, United States

**Keywords:** inducible nitric oxide, lymphatics, lymphedema, nitrosative stress, sex differences

## Abstract

Secondary lymphedema is a common complication following surgical treatment of solid tumors. Although more prevalent in women due to higher breast cancer rates, men also develop lymphedema, often with more severe manifestations. Despite these differences in clinical presentation, the cellular mechanisms underlying sex differences are poorly understood. Previous studies have shown that inducible nitric oxide synthase (iNOS) expression by inflammatory cells is an important regulator of lymphatic pumping and leakiness in lymphedema and that lymphatic endothelial cells are highly sensitive to nitrosative stress. Based on this rationale, we used a mouse tail model of lymphedema to study the role of nitric oxide in sex-related differences in disease severity. Consistent with clinical findings, we found that male mice have significantly worse tail edema and higher rates of tail necrosis compared with female mice following tail skin/lymphatic excision (p = 0.001). Our findings correlated with increased tissue infiltration of iNOS + inflammatory cells, increased iNOS protein expression, and increased nitrosative stress in male mouse lymphedematous skin tissues (p < 0.05). Importantly, transgenic male mice lacking the iNOS gene (iNOS-KO) displayed markedly reduced swelling, inflammation, and tissue necrosis rates, whereas no differences were observed between wild-type and iNOS-KO female mice. Overall, our results indicate that iNOS-mediated nitric oxide production contributes to sex-based differences in secondary lymphedema severity, emphasizing the need to consider sex as a biological variable in lymphedema research.

## 1 Introduction

Despite well-established sex-related differences in the rates and severity of secondary lymphedema, the underlying cellular mechanisms driving these disparities remain largely unexplored. For instance, although more women are diagnosed with secondary lymphedema due to the high prevalence of breast cancer, men are also commonly afflicted after surgical treatment for breast and other solid malignancies, often experiencing a more severe disease presentation (Avila et al., 2023; Cooper-Stanton et al., 2022). Men also exhibit an increased prevalence and severity of secondary lymphedema resulting from filarial infections, a disparity that is at least partially independent of differences in exposure rates to the pathogen (Brabin, 1990).

Recent studies have shown that inflammation is a crucial regulator of the pathophysiology of lymphedema. The infiltration of inflammatory cells leads to an increase in the expression of cytokines and growth factors that inhibit lymphatic endothelial cell proliferation, migration, and differentiation (Zampell et al., 2012; Ly et al., 2017; Brown et al., 2023). Inflammation also increases lymphatic leakiness, impairs lymphatic pumping, and results in dilatation of capillary and collecting lymphatics (Brown et al., 2023). Excessive dilatation of collecting lymphatics leads to valve failure and retrograde flow of lymphatic fluid (Ly et al., 2017; Brown et al., 2023). Increased inflammatory cell expression of inducible nitric oxide synthase (iNOS) and subsequent elevation in perilymphatic nitric oxide also plays an important role in lymphatic function in inflamed tissues by disrupting the intrinsic gradients of endothelial nitric oxide synthase (eNOS) that regulates rhythmic contraction and dilatation of lymphatic collectors (Singla et al., 2022; Rehal et al., 2020; Hagendoorn et al., 2004; Breslin et al., 2018). The increased production of NO further amplifies inflammation and results in the formation of nitrogen and oxygen radicals, thereby increasing nitrosative and oxidative stress. This is important because lymphatic endothelial cells are more vulnerable to nitrosative and oxidative stress, compared with blood endothelial cells (Rehal et al., 2020).

Estrogen contributes to cardiovascular protection in premenopausal women through its anti-inflammatory and vasoprotective properties, as well as its ability to confer resistance to nitrosative and oxidative stress (Iorga et al., 2017; Dragin et al., 2017). Based on this rationale, we tested the hypothesis that NO production resulting from inflammatory cell production of iNOS plays an important role in the pathophysiology of lymphedema and contributes to sex-related disparities in disease presentation. We performed a retrospective analysis of a large, prospectively collected dataset of men and women treated with axillary lymph node dissection (ALND) for breast cancer showed that male sex is an independent risk factor for disease development. To understand the underlying mechanisms, we used a mouse tail model of lymphedema. Consistent with our clinical findings, male mice exhibited a more severe lymphedema phenotype. Furthermore, by comparing wild-type mice with iNOS knockout mice, we showed that increased inflammatory cell expression of iNOS is a major regulator of sex-related differences in lymphedema severity.

## 2 Materials and methods

### 2.1 Clinical data collection and ethics statement

The Institutional Review Board (IRB) of Memorial Sloan Kettering Cancer Center (MSK) approved our retrospective review of breast cancer patients treated at MSK (IRB protocol #18–177). We collected demographic and clinical data from patients with a history of ALND or sentinel lymph node biopsy (SLNB) for the treatment of breast cancer from 1995 to 2022. Demographic variables were age at time of surgery, body mass index (BMI), race, and biological sex. Clinical data included chemotherapy history, radiation history, and breast cancer related lymphedema diagnosis. All variables were collected using a back-end query of the electronic medical record. All patients were deidentified to maintain patient confidentiality. Diagnosis of breast cancer-related lymphedema (BCRL) was determined using ICD9 and 10 codes, specifically I89.0, I97.2, and 457.1.

### 2.2 Mice and ethics statement

The Institutional Animal Care and Use Committee (IACUC) at MSK approved all animal experiments (IACUC protocol # 06–08–018). MSK follows the National Institutes of Health (NIH) Guide for the Care and Use of Laboratory Animals and operates in accordance with the Animal Welfare Act and the Health Research Extension Act of 1985. Adult (8–14-week-old) wild-type C57BL/6 mice and C57BL/6 iNOS mice (iNOS^−/−^) (B6.129P2-*Nos2* ^*tm1Lau*^/J, strain #002609) were purchased from Jackson Laboratories, Bar Harbor, ME, United States.

### 2.3 Tail lymphedema model and tail measurements

Mice were acclimated to our animal care facility for 1–2 weeks. We employed the tail model of lymphedema using our previously published method (Hsu et al., 2021; Jun et al., 2017; Hassanein et al., 2021), which is simple to perform and mimics the histological and inflammatory changes seen in clinical lymphedema (Hassanein et al., 2021). Briefly, we excised 2–3 mm of tail skin circumferentially 2 cm away from the base of the tail. Careful ligation of superficial and deep lymphatic vessels along the lateral tail veins avoided vascular injury. This was verified by observing pooling of Evan’s blue dye at the distal end of the excision. Digital photographs of mice tails were taken at baseline and continued at 1-week intervals postoperatively for 6 weeks. Tail volume measurements were obtained from these photos following our previously published method (Pan et al., 2006) of using ImageJ software (Schneider et al., 2012) to validate 10-mm gaps along the tail length and applying the truncated tail formula to measure weekly tail volume changes:

$$\frac{\pi \left(r1*r2+r2*r3+r3*r4\right)*h}{4}$$

### 2.4 Miles assay

The Miles assay was performed to asses vascular permeability, as previously described (Brash et al., 2018). Briefly, 200 μL of 1% Evans blue dye was injected retro-orbitally into animals 2 weeks after tail lymphatic excision. After 30 min, the mice were euthanized, and tail skin samples 1 cm distal to the incision were collected. The samples were dried overnight at 55° C, and the Evans blue dye was extracted overnight using formamide at the same temperature and quantified by measuring the absorbance at 620 nm.

### 2.5 Histology and immunohistochemical analysis

Histology and immunohistochemistry were performed using our previously described methods (Ly et al., 2019). Briefly, tail tissues were harvested 1 cm distal to the excision site, fixed in 4% paraformaldehyde, and decalcified in EDTA 2 or 6 weeks after surgery. The tissues were then embedded in paraffin and sectioned at a thickness of 5 μm using a microtome. Hematoxylin and eosin staining was performed using Mayer’s hematoxylin and eosin Y solution (Sigma Aldrich, Burlington, MA). The sections were dehydrated using increasing concentrations of ethanol and xylene before mounting with Cytoseal-60 (Eprida, Kalamazoo, MI; cat#8310–4).

For immunofluorescent staining, 5% normal donkey serum was used to block non-specific antibodies for 1 h at room temperature. Primary antibodies were applied to the sections and incubated in 4°C against macrophages (F4/80^+^) (rat; 1:1,000; Abcam, Cambridge, MA; cat #Ab16911), FITC-iNOS (mouse; 1:400; BD Biosciences, San Jose, CA; cat #610330), and CD4 (rabbit; 1:200; Abcam, Cambridge, MA; cat #ab 183,685). The sections were washed three times in phosphate-buffered saline (PBS) for 5 min each, then respective secondary antibodies (1:1,000) were applied to the sections and incubated in the dark at room temperature for 4 h. The sections were washed again, three times in PBS for 5 min each. 4,6-diamidino-2-phenylindole (DAPI; Molecular Probes/Invitrogen, Eugene, OR; cat #D1306) was used as a nuclear marker and incubated for 20 min in the dark. The sections were then washed and mounted using Fluoromount-G (Invitrogen, Waltham, MA; cat# 00–4,958–02) with a cover slip before imaging.

### 2.6 Western blot analysis and biochemical assays

Protein was extracted from tail skin samples 6 weeks after surgery and quantified using the bicinchoninic acid assay method (Walker and Walker, 1996). Western blots were performed in replicates for both male and female mice (n = 6 per group). Primary antibodies against iNOS (rabbit; 1:200; Abcam, Cambridge, MA; cat #Ab3523), malondialdehyde (MDA) (mouse; 1:1,000; ThermoFisher Scientific, Waltham, MA; cat #MA5-27559), and nitrotyrosine (mouse; 1:500; ThermoFisher Scientific, Waltham, MA; cat #32–1900) were used. Equal protein loading was confirmed with glyceraldehyde 3-phosphate dehydrogenase (GAPDH). Relative band density was determined using ImageJ software and served as a measure of relative protein concentration; protein concentration was normalized to GAPDH.

Biochemical assays were performed to quantify oxidative and nitrosative stress using the Lipid Peroxidation (MDA) kit (Abcam, Cambridge, MA; ab118970) and the Griess Reagent kit (Abcam, Cambridge, MA; ab234044). The tissue samples were harvested, and the assays were performed following the manufacturer’s recommendations. We used a microplate reader to detect oxidative stress at 532 nm and nitrosative stress at 540 nm.

### 2.7 Statistical analysis

All statistical analyses were performed using GraphPad Prism software v10.0.3 (GraphPad Prism Software Inc., San Diego, CA). Demographic and clinical characteristics were reported descriptively using means and standard deviations (SD) for continuous variables and counts and percentages for categorical variables. Multivariable logistic regression was performed to model associations between demographic and clinical characteristics and the development of BCRL. Variables included were biological sex, age, race, BMI, and chemotherapy. Goodness of fit was quantified using area under the curve, pseudo-R-squared (Tjur’s), and Hosmer-Lemeshow test. A probability cutoff of 0.5 was selected for the model. For data from our mouse model, differences between groups were assessed using unpaired Student’s *t* tests, and differences between multiple groups were calculated using one -way analysis of variance (ANOVA), with *p* < 0.05 considered significant.

## 3 Results

### 3.1 Male sex is an independent risk factor for BCRL

Our retrospective review identified a total of 32,408 male and female patients who underwent ALND or SLNB at our institution from 1995 to 2022. Table 1 presents patient characteristics for the 1,952 patients included in our odds ratio (OR) regression analysis and the results of our multivariable logistic regression model using sex, race, age, BMI, chemotherapy, and radiation history, with BCRL development as outcome variables. The OR derived represents an association between an exposure or characteristic (e.g., male sex) and outcome (BCRL). An OR of 1 indicates that there is no association between said characteristic and outcome. The average age at time of surgery (SD) was 55.7 (Iorga et al., 2017) years, and the mean BMI was 27.5 (6.1). Data on sex and race were collected from the electronic medical record. As expected, nearly 96% of our cohort were women and only 4.3% were males. Multivariate logistic regression analysis showed that male sex is a significant independent risk factor for BCRL development, with an OR of 1.78 (95% CI = 1.38, 2.29). Said another way, those who identify as male are 1.78 times more likely to develop BCRL than those who identify as female. Consistent with our previous studies (Montagna et al., 2022), we also found Black race (OR = 1.99 [95% CI = 1.75, 2.26]), increased BMI (OR = 1.03 [95% CI = 1.02, 1.03]), and adjuvant chemotherapy (OR = 2.05 [95% CI = 1.83, 2.29]), to be independently associated with an increased risk of BCRL.

**TABLE 1:** Odds ratio regression analysis of patients with BCRL.

Characteristic	N = 1952^1^	Odds ratio	Confidence interval (95%)	P value
Age, years (SD)	55.7 (12)	0.991	[0.99, 1.00]	<0.0001
Male Sex	78 (4.3%)	1.78	[1.38, 2.29]	0.0001
Race				
Black	404 (20.7%)	1.99	[1.75, 2.26]	<0.0001
Asian	69 (3.5%)	0.50	[0.39, 0.64]	<0.0001
Other	30 (1.5%)	0.62	[0.42, 0.89]	0.0146
BMI, kg/m^2^ (SD)	27.5 (6.1)	1.03	[1.02, 1.03]	<0.0001
Neoadjuvant Chemotherapy	276 (14.1%)	1.60	[1.37, 1.87]	<0.0001
Adjuvant Chemotherapy	1,206 (61.7%)	2.05	[1.83, 2.29]	<0.0001

Values are mean (SD); n (%); n = 1952. Comparisons made by logistic regression analysis of patients with BCRL.

### 3.2 Male mice exhibit increased tail edema and lymphatic leakiness

**FIGURE 1:**
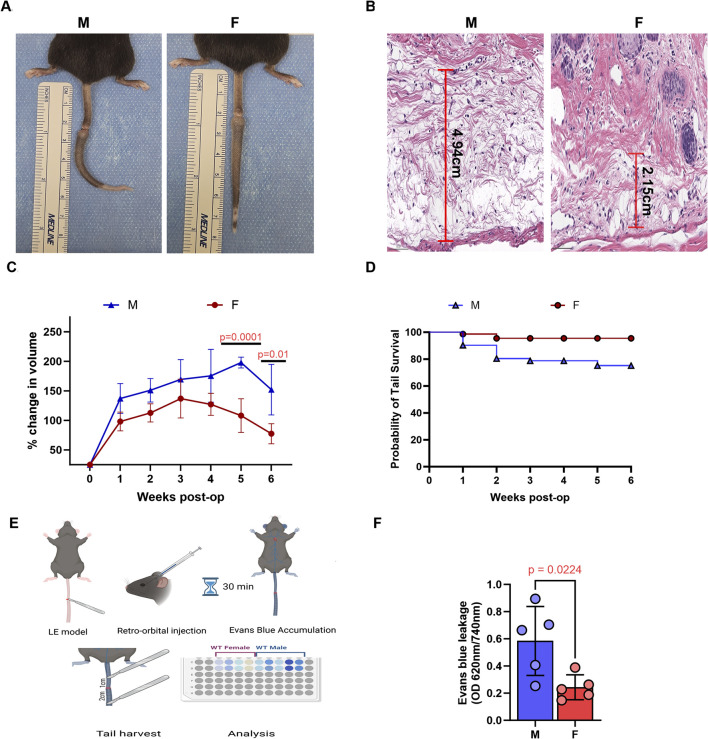
Male mice exhibit more severe tail edema and lymphatic drainage following tail lymphatic excision. **(A)** Digital photograph of mouse tail 6 weeks after tail lymphatic excision (left panel: male; right panel: female). **(B)** Representative H&E of increased edema in male (left panel) compared to female (right panel) mice 1 week after tail lymphatic excision. **(C)** Following tail lymphatic excision, male mice exhibit a greater change in tail volume when compared to female mice undergoing the same surgery (n = 7 per group). Differences observed in tail volume was greatest at 5 and 6 weeks, respectively. **(D)** Kaplan-Meier analysis of tail necrosis rates in male and female mice over a 6-week period. **(E)** Illustration of experimental setup using Evans blue dye to assess lymphatic drainage. **(F)** Quantification of vascular leakage using Evans blue dye absorbance (OD 620 nm/740 nm). N = 5 per group, P values calculated using multiple unpaired t-test. H&E, hematoxylin and eosin; OD, optical density.

Tail lymphatic excision in both male and female mice resulted in tail swelling beginning a few days after surgery (Figure 1A). However, male mice exhibited significantly more edema in tail tissues histologically and increased tail volume grossly compared to females (Figures 1B,C). Male mice also had a much lower tail survival over a 6-week period, as demonstrated by Kaplan-Meir survival analysis (Figure 1D). Notably, many male mice experienced severe tail swelling that resulted in a band-like constriction at the wound site and subsequent tail necrosis distal to the site of skin/lymphatic excision. This effect was independent of surgical technique, as all procedures (for both male and female mice) were performed by the same surgeon across multiple separate experiments. Histologic analysis confirmed increased tissue necrosis in male mice, while evaluation using the Miles assay revealed greater vascular permeability in the tail tissues of male mice compared to female mice (Figures 1E,F).

### 3.3 Inflammation alone does not explain sex differences in lymphedema

**FIGURE 2:**
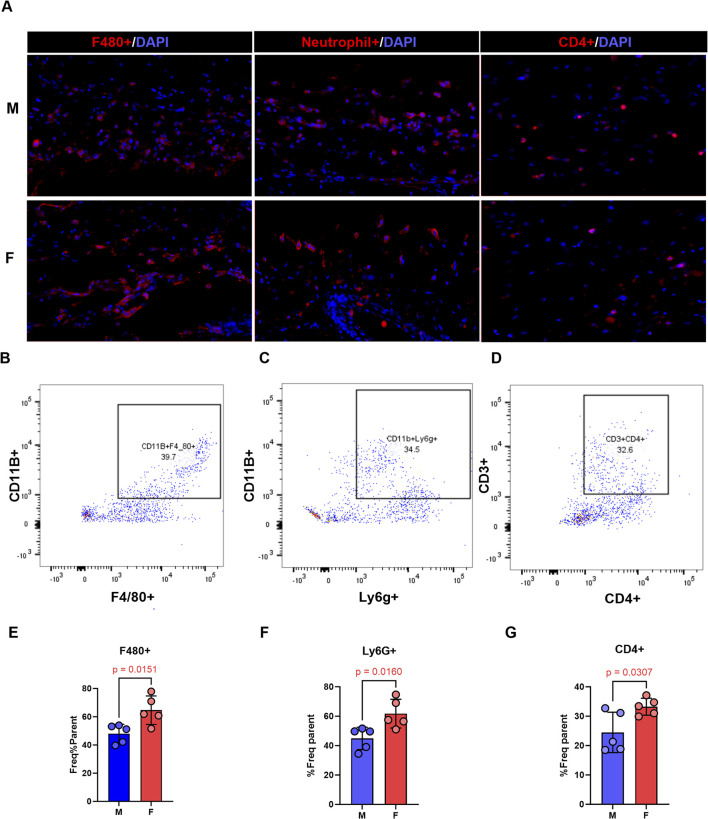
Overall presence of inflammatory cells does not explain worse lymphedema phenotype in male mice. All panels compare male and female mice 2 weeks following tail lymphatic excision. **(A)** Representative immunofluorescence images of inflammatory cells in lymphedema tail skin immunostained with F4/80, neutrophil, or CD4 antibody (scale bar, 20 μm). **(B–D)** Gating pattern, flow cytometry, of inflammatory cells. **(E–G)** Quantitative analysis of inflammatory cells on flow cytometry. N = 4-5 per group, mean ± SD, P values calculated using unpaired t-test.

We compared the accumulation of inflammatory cells in male and female mice 2 weeks after surgery using immunofluorescence and flow cytometry. While qualitative immunofluorescence analysis revealed similar staining areas for macrophages, neutrophils, and CD4^+^ T-cells in both sexes (Figure 2A), flow cytometry analysis of the same tissue showed significantly higher frequencies of all these inflammatory cell populations in female mice (Figures 2B,E–G). This finding was somewhat unexpected considering the decreased edema observed in female mice.

### 3.4 Lymphatic injury in male mice results in increased expression of iNOS and nitrosative stress

**FIGURE 3:**
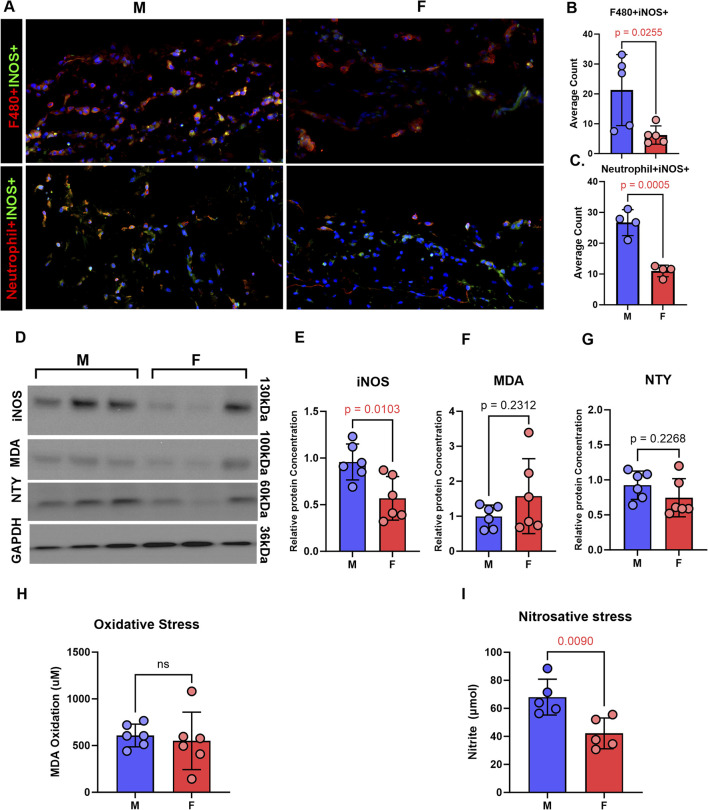
Inducible nitric oxide and nitrosative stress is increased in lymphedema skin in a sex-dependent manner. All panels compare male and female mice 2 weeks following tail lymphatic excision. **(A–C)** Representative immunofluorescence images and quantification (mean ± SD) of inflammatory cells in lymphedema tail skin immunostained for iNOS (scale bar, 20 μm). **(D–G)** Representative postoperative WB and quantification of MDA, nitrotyrosine, and iNOS protein expression in male and female mice. **(H)** Aldehydic lipid peroxidation product, MDA, as a measure of oxidative stress in tail tissue. **(I)** Nitrite production in tail skin as a measure of nitrosative stress. N = 5-6 per group, mean ± SD, P values calculated using unpaired t-test. iNOS, inducible nitric oxide synthase; WB, Western blot; MDA, malondialdehyde.

Immunofluorescence analysis of tail tissues harvested from male and female mice 2 weeks after surgery revealed increased iNOS expression in neutrophils and macrophages in male lymphedema tissues compared to female specimens (Figures 3A–C). Western blot analysis confirmed significantly higher iNOS protein levels in male tail skin (Figures 3D,E). Although trends indicated increased oxidative and nitrosative stress markers (MDA and NTY) in male tissue (Figures 3F,G), only nitrosative stress reached statistical significance based on biochemical analysis (Figure 3I).

### 3.5 iNOS inhibition attenuates lymphedema phenotype to a greater effect in male mice

**FIGURE 4:**
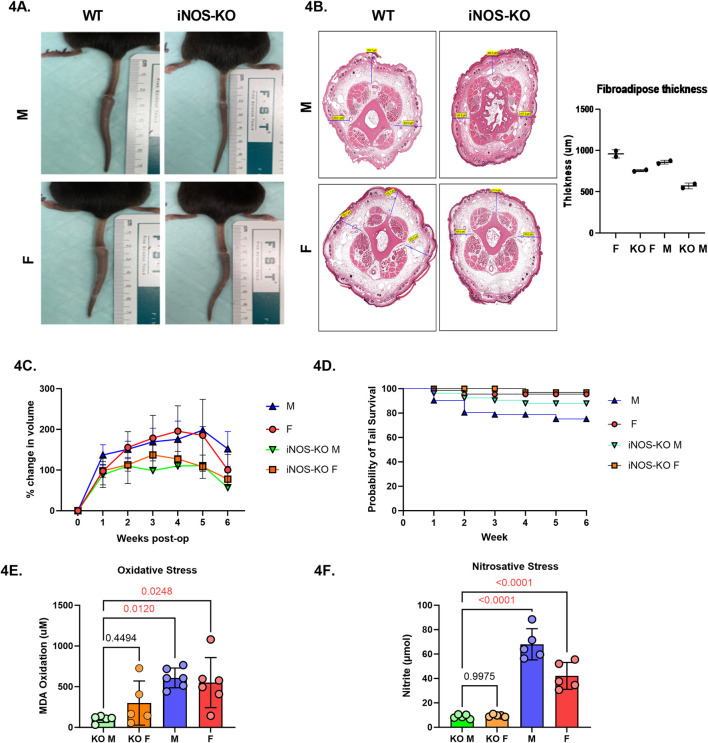
iNOS inhibition attenuates lymphedema phenotype and reduces inflammation in a sex-dependent manner. **(A)** Digital photograph of mouse tail 6 weeks after tail lymphatic excision in WT and iNOS-KO mice (upper panel: male; lower panel; female). **(B)** H&E of fibroadipose deposition at 6-weeks post tail lymphatic excision in male and female iNOS-KO and WT mice. **(C)** Male mice lacking iNOS exhibit a significant reduction in tail volume when compared to WT male mice undergoing tail lymphatic excision (n = 7 per group). Differences observed in tail volume was greatest at 5 weeks. **(D)** Kaplan-Meier analysis of tail necrosis rates in male and female iNOS-KO and WT mice over a 6-week period. **(E)** Aldehydic lipid peroxidation product, MDA, as a measure of oxidative stress in lymphedema tissue when iNOS is deleted. **(F)** Nitrite production in tail skin as a measure of nitrosative stress in lymphedema tissue when iNOS is inactivated. N = 4-5 per group, mean ± SD, P values calculated using one-way ANOVA. WT, wild type; iNOS-KO, inducible nitric oxide synthase-knockout; MDA, malondialdehyde.

To directly assess the role of iNOS in sex-based differences, we utilized iNOS knockout (iNOS-KO) mice. Tail lymphatic excision was performed as before, and tail swelling was monitored weekly for 6 weeks (Figure 4A)). Similarly, histological analysis of subcutaneous thickness of tail cross-sections demonstrated a trend toward greater reduction in fibroadipose thickness in male iNOS-KO mice when compared to wild-type (Figure 4B). Male iNOS-KO mice exhibited significantly reduced tail edema compared to wild-type males, with the most significant difference observed 5 weeks after surgery (Figure 4C, *p* = 0.0033). Female iNOS-KO mice also showed a trend toward lower tail volume increase, however this difference was not statistically significant. Similarly, tail survival was significantly higher in male iNOS-KO mice at 4, 5, and 6 weeks following tail lymphatic excision (Figure 4D) Female mice, both wild-type and KO, had consistently higher tail survival probabilities throughout the study. Notably, biochemical analysis revealed improved overall oxidative and nitrosative stress in both male and female iNOS-KO mice (Figures 4E,F).

## 4 Discussion

Sex-based biological differences significantly influence the progression of numerous prevalent diseases (Cook et al., 2009; Gay et al., 2021; Klein and Flanagan, 2016). Acknowledging this, regulatory bodies such as the U.S. Food and Drug Administration (FDA) and the NIH emphasize the need to consider sex differences in both basic science and clinical research that focus on disease treatment (Duffy et al., 2020). Secondary lymphedema, characterized by progressive inflammation, commonly arises secondary to cancer treatment. BCRL is the most common cause of secondary lymphedema in the United States, with research predominantly concentrating on women, thereby inadvertently marginalizing men, who are frequently excluded (Avila et al., 2023; Co et al., 2020; Reiner et al., 2011). Consequently, male patients often encounter delays in lymphedema diagnosis, potentially leading to exacerbated complications and heightened challenges in treatment (Avila et al., 2023; Cooper-Stanton et al., 2022).

Our study shows that male sex is an independent risk factor for developing BCRL, increasing the disease risk by almost 2-fold. To our knowledge, our analysis includes the largest male cohort with BCRL, and is the first study to identify male sex as a significant variable in lymphedema development. Previous risk studies in BCRL have typically excluded male participants due to the low overall incidence of breast cancer in men (Kim et al., 2013; Aoishi et al., 2020; Voss et al., 2015). In cases of lower extremity lymphedema, reports vary regarding incidence and severity between the two sexes. Some studies note a higher incidence of lower extremity lymphedema in female patients after cancer treatment, while others show that male patients experience more severe lower extremity lymphedema (Gjorup et al., 2017; Thomas and Hamilton, 2014). It is also possible that sex-related differences in lower extremity lymphedema may be related to the high rates of lymphedema resulting from relatively common cancers (e.g., cervical, and vulvar) that exclusively affect women (Dessources et al., 2020; Haidopoulos et al., 2024). Sex disparity in lymphedema resulting from filarial infection is more pronounced, with most studies showing significantly increased (and treatment-resistant) filarial antigen prevalence and higher rates and severity of the disease among male patients (Fimbo et al., 2020; Lau et al., 2014).

Primary lymphedema is a congenital condition that can result from genetic dysfunctions in lymphatic network genes (Senger et al., 2023; Trincot and Caron, 2019; Morfoisse et al., 2021). Although the overall incidence of primary lymphedema is 3.5 times greater among women, men exhibit higher incidences during infancy. Sex differences in primary lymphedema are thought to be attributed to multiple factors. Earlier disease onset in men may be influenced by differential expression of sex hormones between birth and puberty. Particularly, increases in estradiol in women over time is believed to alter this trend from birth to adolescence, as estradiol influences VEGF-C expression, a growth factor implicated in lymphatic development (Senger et al., 2023; Kuonqui et al., 2023). Immune factors also have strong implications in lymphedema pathology (Yuan et al., 2019; Kataru et al., 2019a; David et al., 2016). Altered immune responses in primary lymphatic dysfunction impair lymphangiogenesis and contribute significantly to inflammation, although sex-specific differences in these responses are less explored.

The influence of sex hormones on the vascular system have been extensively studied in cardiovascular diseases (Trincot and Caron, 2019; Willemars et al., 2022; Fontaine et al., 2020). Premenopausal women have lower rates of cardiovascular disease than similar-aged men, indicating hormone-protective effects of estrogens on the arterial vasculature. Indeed, several studies suggest that estrogen directly exerts vasoprotective effects by inducing eNOS-mediated vasodilation in blood endothelial cells (Robert, 2023) and enhancing resistance to oxidative stress in both vascular endothelial and smooth muscle cells (Regitz-Zagrosek and Kararigas, 2017). Like blood endothelial cells, lymphatic endothelial cells express endogenous estrogen receptors (Trincot and Caron, 2019; Morfoisse et al., 2018). In a lymphedema mouse model, Morfoisse et al. reported that estrogen treatment promotes lymphangiogenesis *in vitro* and improves functional lymphatic drainage *in vivo*, suggesting estrogen plays an important role in regulating lymphatic regenerative responses to iatrogenic injury (Morfoisse et al., 2018).

Lymphatic function is altered during inflammation (Schwager and Detmar, 2019; Liao et al., 2011). In our study, we observed slightly increased inflammation at early timepoints on flow analysis in female mice, although this group demonstrated less tail edema and increased tail survival overall. Indeed, there is evidence that estrogens exert anti-inflammatory effects, namely, by suppressing perivascular inflammatory states following vascular injury; however, hormonal fluctuations can influence inflammatory states at various timepoints (i.e., pregnancy and menopause) (Fairweather, 2014; Nasser et al., 2021). Specifically, some studies have shown that estrogen can suppress the production of cytokines responsible for recruiting immune cells like macrophages and neutrophils in certain disease states (Dragin et al., 2017; Vermillion et al., 2018). It is possible that these minor differences result from the effect of estrogen on neutrophils and macrophages—cells that are prevalent and play an important role in the early inflammatory phase of wound healing—since these cells are known to express estrogen receptors (Horng et al., 2017). This hypothesis is supported by the finding that menopausal women with reduced estrogen levels have prolonged inflammatory responses and delayed wound healing.

Obesity-induced lymphatic disfunction results in increased accumulation of iNOS-expressing neutrophils and, to a lesser extent, macrophages (Rehal et al., 2020; Nitti et al., 2016). Increased iNOS activity and nitrosative stress are implicated in various inflammatory and autoimmune diseases, often mediated by macrophages and neutrophils (Sonar and Lal, 2019; Bell et al., 2019; Espey et al., 2000; Zamora et al., 2000). In our study, we noted that male mice have significantly higher levels of iNOS expression than female mice following lymphatic injury. Our study is in line with previous reports showing that male mice have higher iNOS expression in various inflammatory settings (Bell et al., 2019; Genovese et al., 2005; Park et al., 2006). Consistent with our previous studies in obese mice (Torrisi et al., 2016), we found that deletion of iNOS markedly improves lymphatic function and decreases inflammation in male and, to a lesser extent female mice after tail lymphatic ablation. Female mice may be protected from the deleterious effects of iNOS because sex steroids are known to confer protection from reactive oxygen and reactive nitrogen species (Morfoisse et al., 2021; Park et al., 2006; Nofer, 2012).

Neutrophils play an important role in iNOS-induced DNA damage, nitrosative and oxidative stress, and focal ischemia (Espey et al., 2000; Su et al., 2020) (Manda-Handzlik and Demkow, 2015). Lymphatic endothelial cells are particularly sensitive to reactive oxygen and nitrosative stress, with even mild levels causing impaired lymphangiogenesis and lymphatic migration (Singla et al., 2022; Rehal et al., 2020). In addition to causing tissue damage, increased iNOS expression by myeloid cells during inflammatory responses disrupts the endogenous gradients of eNOS, resulting in decreased contraction of lymphatic smooth muscle cells and impaired lymphatic pumping (Liao et al., 2011). Thus, males overall may have increased lymphatic injury and impaired function following injury, and this response may increase the rate and severity of lymphedema.

Our study is subject to several limitations. Firstly, we did not investigate the involvement of other proteins known to mediate pathologic changes in lymphedema, such as TGFβ and PROX-1, in both male and female models (Baik et al., 2022; Kataru et al., 2019b; Ricci et al., 2020). Therefore, we cannot conclusively assert that increased iNOS activity alone accounts for the observed differences. Secondly, the lack of clinical samples for analysis poses a challenge, particularly due to the limited number of male patients undergoing surgery for breast cancer. Expanding our study to encompass various types of lower and upper extremity lymphedema may facilitate a more comprehensive analysis in the future.

In conclusion, we have demonstrated that male mice exhibit more severe tail edema and higher rates of tail necrosis in a tail model of lymphedema compared to females. Additionally, inhibition of iNOS attenuates the lymphedema phenotype and improves nitrosative stress to a greater extent in males. Our findings underscore the significance of biological sex in the development and severity of lymphedema and suggest that inhibition of oxidative or nitrosative stress may have potential as a therapeutic intervention, particularly in males.

## Data Availability

The original contributions presented in the study are included in the article/supplementary material, further inquiries can be directed to the corresponding author.
